# Comparison of Utilization, Costs, and Quality of Medicaid vs Subsidized Private Health Insurance for Low-Income Adults

**DOI:** 10.1001/jamanetworkopen.2020.32669

**Published:** 2021-01-05

**Authors:** Heidi Allen, Sarah H. Gordon, Dennis Lee, Aditi Bhanja, Benjamin D. Sommers

**Affiliations:** 1 Columbia University School of Social Work, New York, New York; 2 Department of Health Law, Policy, and Management, Boston University School of Public Health, Boston, Massachusetts; 3 Department of Health Policy and Management, Harvard T. H. Chan School of Public Health, Boston, Massachusetts; 4 Department of Medicine, Brigham & Women’s Hospital, Boston, Massachusetts

## Abstract

**Importance:**

There has been little rigorous evidence to date comparing public vs private health insurance. With policy makers considering a range of policies to expand coverage, understanding the trade-offs between these coverage types is critical.

**Objective:**

To compare months of coverage, utilization, quality, and costs between low-income adults with Medicaid vs those with subsidized private (Marketplace) insurance.

**Design, Setting, and Participants:**

This cross-sectional study used a propensity score–matched sample of adults enrolled in either Medicaid or Marketplace plans at any point between January 1, 2014, and December 31, 2015. The sample was restricted to individuals with incomes narrowly above and below 138% of the federal poverty level (FPL), which represented the eligibility cutoff between the programs. Data were obtained from 3 state agencies merging comprehensive insurance claims with income eligibility data for Colorado Medicaid expansion and Marketplace enrollees. Income data were linked with an all-payer claims database, and generalized linear models were used to adjust for clinical and demographic confounders. Participants included 8182 low-income nonpregnant adults aged 19 to 64 years enrolled in Medicaid or Marketplace coverage during the 2014 to 2015 period, with incomes between 134% and 143% of the FPL.

**Exposures:**

Health insurance through Colorado Medicaid or Colorado’s state-based Marketplace.

**Main Outcomes and Measures:**

The primary analytical approach was a multivariate regression analysis of the propensity score–matched sample. Primary outcomes were months of coverage in Medicaid or Marketplace insurance, office and emergency department (ED) visits, ambulatory care–sensitive hospitalizations, and total costs. For secondary quality outcomes, the propensity score–matched sample was widened to 129% to 148% of the FPL to ensure adequate sample size. Secondary outcomes included prescription drug utilization, types of ED visits, hospitalizations, out-of-pocket costs, and clinical quality measures. Primary data analysis was between September 2018 to July 2019, with revisions finalized in November 2020.

**Results:**

The propensity score–matched narrow-income sample included a total of 8182 participants (4091 Medicaid eligible [50%]: mean [SD] age, 42.8 [13.6] years; 2230 women [54.5%]; 4091 Marketplace eligible [50%]: mean [SD] age, 42.7 [13.9] years; 2229 women [54.5%]). Demographic differences across the 2 groups were well balanced, with all standardized mean differences less than 0.10. Marketplace coverage was associated with fewer ED visits (mean, 0.36 [95% CI, 0.32-0.40] visits vs 0.56 [95% CI, 0.50-0.62] visits; *P* < .001) and more office (outpatient) visits than Medicaid (mean, 2.22 [95% CI, 2.11-2.32] visits vs 1.73 [95% CI, 1.64-1.81] visits; *P* < .001). No differences in ambulatory care–sensitive hospitalizations were found (0.004 [95% CI, 0.001-0.006] vs 0.007 [95% CI, 0.002-0.011]; *P* = .15). Total costs were 83% higher in Marketplace coverage (mean, $4553 [95% CI, $3368-$5738] vs $2484 [95% CI, $1760-$3209]; *P* < .001) owing almost entirely to higher prices, and out-of-pocket costs were 10 times higher (mean, $569 [95% CI, $337-$801] vs $45 [95% CI, $26-$65]; *P* < .001). Five of 12 secondary quality measures favored private insurance, and 1 favored Medicaid.

**Conclusions and Relevance:**

In this cross-sectional propensity score–matched study, Medicaid and Marketplace coverage differed in important ways. Public coverage through Medicaid was associated with more ED visits and fewer office visits than private Marketplace coverage, which may reflect barriers to outpatient care or lower cost-sharing barriers to ED care in Medicaid. Results suggest that Medicaid coverage was substantially less costly to beneficiaries and society than private coverage, with mixed results on health care quality.

## Key Points

**Question:**

How do utilization, cost, and quality compare between public (Medicaid) and private (Marketplace) health insurance?

**Findings:**

This cross-sectional study of 8182 participants used a propensity score–matched sample narrowed to 5 percentage points above and below the federal poverty level threshold that separates Medicaid and Marketplace eligibility (138%). Marketplace coverage was associated with fewer emergency department visits and more office visits than Medicaid, total costs were 83% higher in Marketplace coverage owing to much higher prices, and out-of-pocket spending was 10 times higher in Marketplace coverage; results for quality of care were mixed.

**Meaning:**

This study found that Medicaid and Marketplace coverage differ in important ways: more emergency department visits in Medicaid may reflect impaired access to outpatient care or lower copayments; Marketplace coverage was more costly owing to higher prices and also had higher cost sharing for consumers.

## Teaser

This cross-sectional study uses data from 3 state agencies merging comprehensive insurance claims with income eligibility data for Colorado Medicaid expansion and Marketplace enrollees to investigate health care utilization, cost, and quality between public and private health insurance for low-income adults.

## Introduction

Numerous studies over the past decade have evaluated the effects of health insurance on utilization, costs, and quality, and most of this evidence comes from research on Medicaid.^1,2,3,4^ Yet there has been little rigorous evidence on the comparative effects of public vs private coverage.

The primary reason for this literature gap is that individuals with private insurance differ substantially from individuals with public insurance. Medicaid covers lower-income individuals, many of whom are disabled and unable to work, whereas most adults with private coverage obtain insurance through employment. Flawed observational studies have suggested that Medicaid leads to worse health outcomes compared with private insurance^5,6,7^ or even having no insurance.^8^ But these studies share a critical weakness: the inability to adequately control for individual socioeconomic and clinical confounders.^9^ Typically, studies that have used administrative or claims data cannot account for income, one of the key drivers for differences between public and private insurance, and survey-based analyses lack detailed clinical information.^10,11^ Our study overcomes these limitations through a novel data set combining all-payer claims data and income-based eligibility information.

Understanding the trade-offs between public and private coverage is critical for policy makers. Several states are proposing or implementing Medicaid expansions that attempt to align Medicaid with private insurance (such as higher cost sharing) or feature partial expansions in which some low-income individuals enroll in Marketplace coverage instead.^12,13^ Other states are considering a Medicaid buy-in option.^14^ President-elect Joe Biden has proposed a public option for coverage, whereas other Democrats advocate for Medicare-for-All plans that would replace private insurance with public coverage.^15^

Given this policy context, our objective was to leverage a unique data source and the natural experiment created by the Patient Protection and Affordable Care Act (ACA)’s income-based insurance expansion to analyze differences in utilization, costs, and quality among low-income adults with public vs private coverage.

## Methods

### Study Design

Our cross-sectional study used a propensity score–matched sample to compare utilization, quality of care, and costs for individuals with Medicaid vs subsidized Marketplace insurance, with coverage determination based on the income eligibility threshold of 138% of the federal poverty level (FPL). Under the ACA, this threshold sorts the low-income population lacking employer-sponsored insurance into 2 coverage types: Medicaid at or below 138% of the FPL, and subsidized Marketplace coverage above 138% of the FPL. This project was approved by the Harvard T. H. Chan School of Public Health’s institutional review board. As this study used preexisting secondary data for analysis, informed consent was waived. This study followed the Strengthening the Reporting of Observational Studies in Epidemiology (STROBE) reporting guideline.

To compare individuals with similar incomes but different coverage types, we limited the Medicaid-eligible and Marketplace-eligible samples within 5 percentage points of the FPL cutoff. This approach conceptually resembles a regression discontinuity design, which examines changes in outcomes just above and below a sorting variable threshold.^16^ However, enrollment in Medicaid and Marketplace coverage is optional, and the latter requires a premium, creating nonrandom selection that may be influenced by underlying health and the need for care. To address this, we used propensity score matching based on demographic and clinical features to compare public and private coverage while minimizing confounding.^9,11^

### Data

We obtained a unique data set from 3 state agencies, merging comprehensive insurance claims with income eligibility data for Colorado Medicaid expansion and Marketplace enrollees from January 1, 2014, to December 31, 2015 (eMethods in the Supplement). The Colorado All-Payer Claims Database contains data on insurance enrollment; utilization and payments for outpatient, inpatient, and prescription drugs; and beneficiary demographic details. Family income data collected at enrollment (as a percentage of the FPL) were provided by the state. The earliest measurement of family income in each calendar year was used to define eligibility, consistent with an intent-to-treat approach.

### Sample Construction

The initial unmatched sample included adults aged 19 to 64 years with at least 1 month of Medicaid (via the ACA eligibility expansion) or Marketplace coverage at any point in 2014 or 2015, with incomes between 75% and 400% of the FPL. We created our primary study sample using a propensity score match within a narrow-income band 5 percentage points below and above the 138% eligibility income eligibility cutoff. The sample excluded pregnant women, who have different eligibility criteria for Medicaid, and individuals with incomes below 138% of the FPL in subsidized Marketplace coverage (primarily individuals ineligible for Medicaid based on immigration status).

Within this income range, we implemented a 1:1 propensity score match for having a Marketplace-eligible income. The propensity score model included age, sex, urban-rural status (based on 3-digit zip codes), Elixhauser Comorbidity Index,^17^ and the 5 most prevalent conditions in the sample: hypertension, depression, chronic obstructive pulmonary disorder, hypothyroidism, and diabetes (types 1 and 2). Substance abuse was not calculated in the Elixhauser Comorbidity Index because claims with this diagnosis were generally excluded by the state in constructing the all-payer claims database based on federal regulations. We did not include race/ethnicity because it is not reliably captured across the Marketplace and Medicaid data (eMethods in the Supplement).

### Outcome Measures

Our outcome domains were coverage, utilization, cost, and quality. Coverage outcomes were months of Medicaid, months of Marketplace, and months of either type of coverage. Primary utilization outcomes were the number of outpatient and ED visits. Secondary utilization outcomes were number of prescription drug fills and hospitalizations.

For costs, the primary outcome was the total cost of care across all claims (combining out-of-pocket and insurer-paid costs). Secondary outcomes were out-of-pocket costs (the amount charged to patients after accounting for Marketplace cost-sharing reductions) and price-normalized spending (with each billed service adjusted to average Medicaid prices to filter out price differences by insurance type).^18^

The primary quality outcome was the number of ambulatory care–sensitive hospitalizations.^19^ For secondary outcomes, we measured annual rates of influenza vaccination; mammograms (women aged 50-64 years)^20^; chlamydia screening (women aged 19-24 years)^21^; β-blocker use (patients with coronary artery disease)^22^; statin use (patients with diabetes older than 40 years or those with atherosclerotic disease)^23^; and hemoglobin A_1c_ testing, eye examinations,^24^ and urine microalbumin testing (patients with diabetes).^25^ We also examined several measures of low-value care: advanced imaging for uncomplicated back pain or headaches, narcotic prescriptions for headaches, and antibiotic prescriptions for upper respiratory infections (eMethods in the Supplement).^26^

### Statistical Analysis

The primary analytical approach was a multivariate regression analysis of the propensity score–matched sample. We used a generalized linear model with the following distributions: negative binomial distribution for months of coverage, utilization measures, and ambulatory care–sensitive admissions to account for overdispersion in the underlying count data; binomial distribution with a logit link for binary clinical quality metrics; and gamma distribution and a log link for costs.^27^ For each outcome, we present adjusted rates for Medicaid vs Marketplace using predicted marginal effects.

The independent variable of interest was an indicator for Marketplace eligibility (income >138% FPL) vs Medicaid eligibility (≤138% FPL). Models controlled for age, sex, calendar year, Elixhauser Comorbidity Index, the 5 most common clinical conditions listed above, and 3-digit zip code. In models examining utilization, quality, and costs, we controlled for total months of coverage with similar results.

For secondary quality outcomes, most of which used a clinical subset of patients, we widened the propensity score–matched sample to 129% to 148% of the FPL to ensure adequate sample size, and then the specific clinical subsets were drawn based on exclusion criteria from that single matched sample. Due to the risk of overfitting, we also replaced the 3-digit zip codes with urban vs rural residence for these regressions.

For all outcomes, we calculated per-comparison *P* values and prespecified family-wise adjusted *P* values within the outcome domains of coverage, utilization, cost, and quality to account for multiple comparisons. We used the free step-down resampling method of Westfall and Young,^28^ which accounts for the probability of falsely rejecting at least 1 true null hypothesis in each domain.^29^

We conducted several sensitivity analyses (eAppendix in the Supplement). First, we analyzed each year (2014 and 2015) separately to test whether the results varied over time. Second, we assessed the effect of excluding claims from the first month of coverage, because Medicaid enrollment (unlike Marketplace coverage) sometimes occurs retroactive to a hospital or emergency department (ED) visit, which could influence utilization and costs. Similarly, we assessed a model excluding all individuals whose first claim in the year was an ED- or hospital-based visit in their first month of coverage.

We also analyzed changes in the types of ED visits using an updated version of the New York University algorithm.^30,31^ Finally, we used a broader 129% to 148% FPL income range for our matched sample.

*P* < .05 was considered significant, and all *P* values were 2 sided. Primary data analysis was performed between September 2018 and July 2019, with revisions finalized in November 2020, using STATA, version 14.0 (StataCorp LLC).

## Results

### Sample Characteristics and Matching

The propensity score–matched narrow-income sample included a total of 8182 participants (4091 Medicaid eligible [50%]: mean [SD] age, 42.8 [13.6] years; 2230 women [54.5%]; 4091 Marketplace eligible [50%]: mean [SD] age, 42.7 [13.9] years; 2229 women [54.5%]). Table 1 shows descriptive statistics for the unmatched sample and the propensity score–matched sample. Demographic differences across the 2 groups were well balanced, with all standardized mean differences less than 0.10. After matching, the Medicaid-eligible group had a mean income of 136% FPL, whereas the Marketplace group had a mean income of 141% FPL (approximately $16 000 vs $16 600 annually for an individual or $27 300 vs $28 300 for a family of 3, as of 2015). In the unmatched sample (n = 470 417), individuals in Medicaid (75%-138% FPL) were on average younger, disproportionately women, and more urban than those with Marketplace coverage (370 739 Medicaid eligible [78.8%]: mean [SD] age, 38.0 [12.1] years; 223 041 women [60.2%]; 99 678 Marketplace eligible [21.2%]; mean [SD] age, 45.7 [13.3] years; 54 217 women [54.4%]; 138%-400% FPL).

**Table 1.** Descriptive Statistics for the Study Sample

Characteristic	Full sample (income 75%-400% FPL)^a^			Propensity score–matched sample (income 134%-143% of FPL)		
	No. (%)^b^		SMD	No. (%)		SMD
	Medicaid eligible (≤138% FPL) (n = 370 739)	Marketplace eligible (>138% FPL) (n = 99 678)		Medicaid eligible (≤138% FPL) (n = 4091)	Marketplace eligible (>138% FPL) (n = 4091)	
Average income, % FPL	106%	222%		136%	141%	NA
Matching variables						
Mean (SD) age, y	38.0 (12.1)	45.7 (13.3)	0.607	42.8 (13.6)	42.7 (13.9)	0.004
19-25	60 133 (16.2)	7629 (7.7)	0.244	563 (13.8)	569 (13.9)	0.004
26-34	109 683 (29.6)	18 572 (18.6)	0.246	842 (20.6)	844 (20.6)	0.001
35-44	91 430 (24.7)	17 244 (17.3)	0.175	691 (16.9)	686 (16.8)	0.003
45-54	61 709 (16.6)	21 474 (21.5)	0.128	866 (21.2)	857 (21.0)	0.005
55-64	47 784 (12.9)	34 759 (34.9)	0.578	1129 (27.6)	1135 (27.7)	0.003
Sex						
Men	147 584 (39.8)	45 402 (45.6)	0.117	1859 (45.4)	1861 (45.5)	0.003
Women	223 041 (60.2)	54 217 (54.4)	0.117	2230 (54.5)	2229 (54.5)	0.002
Rural area of residence	30 761 (8.3)	12 268 (12.3)	0.139	458 (11.2)	458 (11.2)	0.000
Elixhauser Comorbidity Index (score)	0.32	0.29	0.015	0.22	0.25	0.013
Most common chronic conditions						
Hypertension	17 213 (4.6)	4917 (4.9)	0.014	178 (4.4)	179 (4.4)	0.001
Depression	20 054 (5.4)	3918 (3.9)	0.067	170 (4.2)	181 (4.4)	0.016
COPD	15 693 (4.2)	2630 (2.6)	0.082	127 (3.1)	129 (3.2)	0.004
Hypothyroidism	10 097 (2.7)	3258 (3.3)	0.033	114 (2.8)	113 (2.8)	0.004
Diabetes	16 061 (4.3)	3881 (3.9)	0.022	165 (4.0)	162 (4.0)	0.004
Selected high-cost conditions						
Congestive heart failure	1362 (0.4)	328 (0.3)	0.007	9 (0.2)	9 (0.2)	0.000
AIDS/HIV	511 (0.1)	374 (0.4)	0.055	9 (0.2)	11 (0.3)	0.010
Lymphoma	328 (0.1)	115 (0.1)	0.01	6 (0.2)	1 (0.0)	0.044
Metastatic cancer	634 (0.2)	203 (0.2)	0.007	1 (0.0)	4 (0.1)	0.032
Solid tumor without metastasis	3367 (0.9)	1414 (1.4)	0.051	37 (0.9)	46 (1.1)	0.022
Rheumatoid arthritis or collagen vascular	3312 (0.9)	984 (1.0)	0.011	28 (0.7)	42 (1.0)	0.038

Abbreviations: COPD, chronic obstructive pulmonary disease; FPL, federal poverty level; NA, not applicable; SMD, standardized mean difference (absolute value of difference in means divided by the standard deviation).

^a^ Sample from Colorado All-Payer Claims Database, linked to income data from Medicaid and Marketplace eligibility files.

^b^ Values are written as No. (%) unless otherwise stated.

### Coverage Outcomes

**Figure 1.**
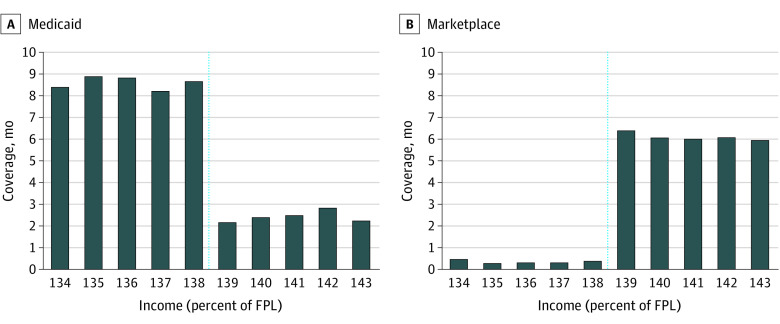
Months of Medicaid or Marketplace Coverage per Year, by Income as a Percentage of the Federal Poverty Level (FPL) Data are from the Colorado All-Payer Claims Database, linked to income data from A, Medicaid and B, Marketplace eligibility files. Sample contains propensity score–matched adults aged 19 to 64 years, with incomes between 134% and 143% of FPL (N = 8182). Income was measured as the first recorded value in each calendar year. Some individuals subsequently experienced changes in income later in the year, but the results above reflect an intent-to-treat analysis, leading to some months of coverage in Medicaid among those whose income was originally >138% of FPL and some months of coverage in Marketplace plans among those whose income was originally ≤138% of FPL. The dashed line indicates the Medicaid income eligibility threshold at 138% of FPL.

Figure 1 and Table 2 display months of coverage for the 2 groups. We found strong sorting into Medicaid and Marketplace coverage types based on the ACA’s income threshold. Overall months of coverage—conditional on enrollment—were fairly similar, with Medicaid associated with roughly 2 additional weeks of coverage per year (mean, 8.90 [95% CI, 8.78-9.01] months vs 8.48 [95% CI, 8.37-8.60] months; *P* < .001).

**Table 2.** Differences in Coverage and Utilization Between Those Eligible for Medicaid vs Marketplace Insurance^a^

Outcome	Adjusted mean (95% CI)		Public vs private difference	
	Medicaid eligible (income 134% to ≤138% FPL)	Marketplace eligible (income >138% to ≥143% FPL)	*P* value	Adjusted *P* value^b^
Coverage, mo^c^				
Medicaid or Marketplace	8.90 (8.78-9.01)	8.48 (8.37-8.60)	<.001	<.001
Medicaid	8.67 (8.53-8.82)	2.27 (2.14-2.39)	<.001	<.001
Marketplace^c^	0.34 (0.30-0.40)	5.65 (5.47-5.84)	<.001	<.001
Utilization (per year), No.				
Outpatient visits	1.73 (1.64-1.81)	2.22 (2.11-2.32)	<.001	<.001
Emergency department visits	0.56 (0.50-0.62)	0.36 (0.32-0.40)	<.001	<.001
Prescription drug fills	7.41 (6.84-7.97)	8.29 (7.66-8.93)	.02	.05
Hospitalizations	0.032 (0.021-0.043)	0.028 (0.018-0.038)	.47	.49

Abbreviations: FPL, federal poverty level; GLM, generalized linear model.

^a^ Data are from the Colorado All-Payer Claims Database, linked to income data from Medicaid and Marketplace eligibility files. Sample contains propensity score–matched adults aged 19 to 64 years, with incomes between 134% and 143% of FPL (N = 8182). Models adjust for age, sex, Elixhauser Comorbidity Index (overall score and top 5 conditions), year, and 3-digit zip code; utilization outcomes also adjust for total months of Medicaid or Marketplace coverage. Coverage and utilization outcomes were analyzed using a GLM with a negative binomial distribution. All regression results were converted to adjusted means based on the observed distribution of covariates using the margins command in STATA, other than for coverage outcomes as noted.

^b^ These *P* values were adjusted according to the family-wise error rate, using the Westfall and Young^28^ free step-down resampling approach, to account for multiple outcomes within each category.

^c^ Months of Medicaid and months of Marketplace do not sum to the composite outcome of months covered because 0.6% of respondents lacked data on the coverage type for a given month, but we still included these months of coverage in our primary outcome. Coverage outcomes were assessed using margins at covariate means, due to totaling errors with the margins command at the observed distribution (ie, total months of coverage < months Medicaid).

### Utilization Outcomes

Table 2 also presents utilization results. Adults eligible for Medicaid had 0.49 fewer outpatient visits (mean, 1.73 [95% CI, 1.64-1.81] visits vs 2.22 [95% CI, 2.11-2.32] visits; *P* < .001) and 0.20 more ED visits (mean, 0.56 [95% CI, 0.50-0.62] visits vs 0.36 [95% CI, 0.32-0.40] visits; *P* < .001) per year than Marketplace-eligible adults. Adults eligible for Marketplace had 0.88 more prescriptions filled (mean, 8.29 [95% CI, 7.66-8.93] prescriptions vs 7.41 [95% CI, 6.84-7.97] prescriptions; *P* = .02) per year than Medicaid-eligible adults. There were no significant differences in number of hospitalizations.

### Costs and Quality Outcomes

Table 3 presents costs and quality differences. Mean annual spending was $2484 (95% CI, $1760-$3209) among Medicaid-eligible adults and $4553 (95% CI, $3368-$5738) among Marketplace-eligible adults (*P* < .001). Out-of-pocket costs averaged $45 (95% CI, $26-$65) per enrollee per year in Medicaid compared with $569 (95% CI, $337-$801) in Marketplace (*P* < .001). Normalized total costs showed no significant differences by coverage type.

**Table 3.** Differences in Health Care Costs and Quality Between Those Eligible for Medicaid vs Marketplace Insurance With Cost-Sharing Reductions^a^

Outcome	Adjusted mean (95% CI)		Public vs private difference	
	Medicaid eligible (income 134%-≤138% FPL)	Marketplace eligible (income >138%-≤143% FPL)	*P* value	Adjusted *P* value^b^
Cost, $				
Total health care	2484 (1760-3209)	4553 (3368-5738)	<.001	<.001
Out of pocket^c^	45 (26-65)	569 (337-801)	<.001	<.001
Normalized spending, using mean Medicaid prices^d^	1490 (1215-1765)	1642 (1379-1905)	.17	.31
Quality				
Ambulatory care–sensitive hospitalizations	0.007 (0.002-0.011)	0.004 (0.001-0.006)	.15	.18

Abbreviations: FPL, federal poverty level; GLM, generalized linear model.

^a^ Data are from the Colorado All-Payer Claims Database, linked to income data from Medicaid and Marketplace eligibility files. Sample contains propensity score–matched adults aged 19 to 64 years, with incomes between 134% and 143% of FPL (N = 8182). Models adjust for age, sex, Elixhauser Comorbidity Index (overall score and top 5 conditions), year, 3-digit zip code, and total months of Medicaid or Marketplace coverage. Costs outcomes were analyzed using a GLM with a gamma distribution and log link, with outcomes in 2015 inflation-adjusted terms. Quality outcomes were analyzed using a GLM with a negative binomial distribution. All regression results were converted to adjusted means based on the observed distribution of covariates using the margins command in STATA.

^b^ These *P* values were adjusted according to the family-wise error rate, using the Westfall and Young^28^ free step-down resampling approach, to account for multiple outcomes within each category.

^c^ Out-of-pocket costs are the charged amount; the data set does not indicate whether patients paid the required amount.

^d^ This outcome was calculated using mean Medicaid price per service provided, to provide an aggregate measure of health care resources consumed but using the same price regardless of the person’s type of health insurance (eMethods in the Supplement).

**Figure 2.**
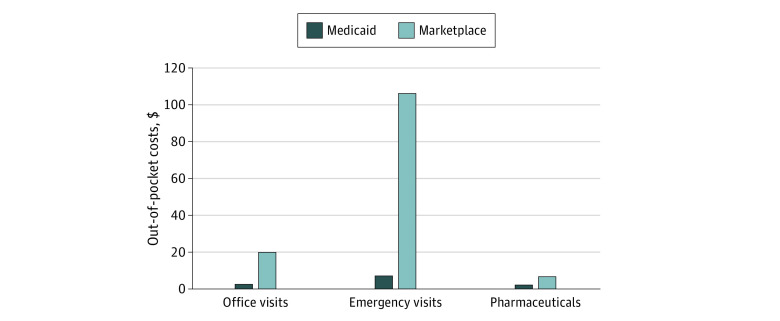
Average Out-of-Pocket Costs per Visit or Prescription Among Adults Eligible for Medicaid vs Marketplace Coverage With Cost-Sharing Reductions Data are from the Colorado All-Payer Claims Database, linked to income data from Medicaid and Marketplace eligibility files. Sample contains propensity score–matched adults aged 19 to 64 years, with incomes between 134% and 143% of the federal poverty level (FPL) (N = 8182). Results reflect an intent-to-treat analysis based on the first measured income value in each calendar year. Medicaid eligible reflects those with initial incomes ≤138% of FPL, whereas Marketplace eligible reflects those with initial incomes >138% of FPL. Out-of-pocket costs are the charged amount; the data set does not indicate whether patients paid the required amount.

Figure 2 presents descriptive results comparing average out-of-pocket costs per claim for different types of utilization. Per-visit and per-prescription cost sharing was significantly higher in Marketplace coverage than Medicaid ($20.29 vs $2.80 for office visits, $106.21 vs $7.27 for emergency visits, and $6.82 vs $2.40 per prescription; all *P* < .001), even with federal cost-sharing reductions. There was no significant difference between Medicaid-eligible and Marketplace-eligible adults in the number of ambulatory care–sensitive hospitalizations (mean, 0.007 [95% CI, 0.002-0.011] vs 0.004 [95% CI, 0.001-0.006]; *P* = .15).

### Sensitivity Analyses

Our results for 2014 vs 2015 were largely similar, with a few exceptions (eTable 1 in the Supplement). The total months of coverage differed significantly in 2015 but not 2014, whereas prescription drug fills were significantly greater for Marketplace coverage than Medicaid in 2015 only (mean, 8.26 [95% CI, 7.37-9.14] vs 5.86 [95% CI, 5.20-6.51]; *P* < .001).

After excluding the first month of claims for each person in each year (eTable 2 in the Supplement), the gap in ED visits between Medicaid and Marketplace coverage shrank by approximately 30% (0.13 additional visits per year instead of 0.20), whereas spending was significantly higher in Marketplace coverage than in Medicaid even after normalization to Medicaid prices (mean, $1557 [95% CI, $1303-$1811] vs $1283 [95% CI, $1038-$1528]; *P* = .008). Excluding from the sample all individuals whose first claim in a given year was in the ED or hospital (n = 182) produced similar results as excluding the first month of claims from all respondents (eTable 3 in the Supplement).

Higher ED visit rates in Medicaid than Marketplace coverage were present across most major visit types, including emergent nonpreventable visits (0.11 vs 0.07; *P* < .001), emergent but preventable visits (0.04 vs 0.03; *P* = .001), primary care treatable visits (0.13 vs 0.08; *P* < .001), and nonemergent visits (0.12 vs 0.08; *P* < .001) (eTable 4 in the Supplement).

A propensity score–matched sample using a wider income range (from 129%-148% FPL) achieved good balance on demographic and clinical features (eTable 5 in the Supplement) and produced similar results as the primary model (eTable 6 in the Supplement).

The larger sample also enabled us to assess several secondary quality outcomes (eTable 7 in the Supplement). Five of the high-value care measures were significantly higher for Marketplace coverage (mammograms, chlamydia screening, hemoglobin A_1c_, and urine microalbumin testing among diabetic patients, and influenza vaccination), 1 was significantly higher for Medicaid (β-blocker use for coronary artery disease), and 2 did not differ significantly. None of the 4 low-value care measures differed significantly by coverage type. Statistical significance was similar for per-comparison and family-wise *P* values, except for β-blocker use.

## Discussion

In this cross-sectional analysis of a propensity score–matched sample of low-income adults enrolled in health insurance, we found notable differences in health care utilization and costs between Medicaid and Marketplace coverage, with more modest differences in health care quality. Several patterns in health care utilization emerged. Medicaid was associated with fewer outpatient visits and prescriptions but more ED visits than Marketplace coverage. There are several possible explanations. One possibility is that limited access to outpatient care in Medicaid led some individuals to pursue ED care instead. One recent study reported Medicaid enrollees experienced longer wait times for appointments than those in Marketplace coverage,^32^ and lower physician participation rates in Medicaid is a long-standing concern.^33^ Another potential explanation is that some individuals signed up for Medicaid while in the ED, because Medicaid’s policy of retroactive eligibility creates an additional pathway for people to gain coverage; this option is not available for Marketplace coverage. Our analysis excluding claims from the first month of coverage supported this explanation, as roughly 30% of the higher ED usage in Medicaid occurred in the first month of enrollment.

Finally, the difference in ED utilization was most likely driven in part by the substantial differences in cost sharing between Medicaid and Marketplace plans. Medicaid eligibility was associated with $7.27 in average out-of-pocket costs per ED visit, compared with $106.21 in Marketplace coverage. Our finding that both emergent and nonemergent ED visit rates were higher in Medicaid suggests that a portion of these additional visits were medically necessary. In some cases, lower ED visit rates in Marketplace coverage could potentially reflect sick patients avoiding needed emergency care owing to cost-sharing barriers.

Meanwhile, higher prescription drug counts among those with Marketplace coverage—despite higher drug copays—most likely reflects more outpatient visits and opportunities to receive prescriptions. Whether these differences produce downstream health effects is an important topic for future research.

Turning to costs, overall health care spending was more than 80% higher among Marketplace-eligible adults than among Medicaid-eligible adults. This difference was no longer significant when claims were adjusted to Medicaid prices, indicating that the cost differences were driven by higher prices for the same services in the Marketplace compared with Medicaid.

Marketplace coverage was also associated with 10-fold higher out-of-pocket costs for low-income enrollees than Medicaid. This finding is consistent with prior research that found that Marketplace enrollees are exposed to higher out-of-pocket costs and are at greater risk of extremely high spending even with significant federal subsidies.^10,32,34,35^

In terms of clinical quality, we found no difference for the primary outcome of ambulatory care–sensitive hospitalizations. Among secondary outcomes, 5 measures favored Marketplace coverage (though 1 was of minimal clinical relevance, a 1 percentage-point difference in flu vaccination), 1 measure favored Medicaid, and the rest (6 of 12) showed no significant differences. Lower rates of mammography and chlamydia testing in Medicaid are consistent with a previous analysis, though that study lacked individual-level measures of socioeconomic status.^36^ Results for chronic disease measures varied, with higher rates of hemoglobin A_1c_ and urine microalbumin testing among diabetic patients in Marketplace coverage but higher rates of β-blocker prescription for coronary artery disease in Medicaid. Our finding of similar rates of low-value care in both insurance types was consistent with a prior study.^26^ Overall, our results suggest a mixed picture on quality, with a plurality of secondary outcomes favoring private coverage, but with more than half of the primary and secondary measures showing no significant differences, similar to a prior quasi-experimental study comparing Kentucky’s Medicaid expansion to Arkansas’ private insurance expansion for low-income adults.^34^

### Limitations

Our study has important limitations. First, based on the unique data required for this assessment (simultaneous Medicaid and Marketplace expansion, a state-based exchange for income data at enrollment, and an established all-payer claims database), we analyzed a single state in the US, which may limit generalizability. eTable 8 in the Supplement presents several key characteristics for the state of Colorado compared to the US as a whole. Most notably, Colorado has lower Medicaid managed care participation than most states, but for most other Medicaid and Marketplace features, as well as uninsured rate and median income, Colorado is reasonably close to the national average.^37,38,39^

Second, our study design required us to match income data from Marketplace coverage and Medicaid to all-payer claims data. With Medicaid, we were able to match on a unique Medicaid identifier. For Marketplace, we used probabilistic matching that yielded a match for more than 86% of enrollees; unmatched claims may have introduced bias.

Third, limitations of the data set precluded analysis of other worthwhile topics, including racial/ethnic disparities, the types of physicians providing care, and differences across plan types within Marketplace coverage and Medicaid managed care. Also, substance abuse–related claims in Medicaid and Marketplace coverage were largely absent from the data set based on federal regulations; however, this absence did not preclude individuals with substance abuse claims from study inclusion.^40^

Finally, our analysis was observational. Although we used a narrow band of income and propensity score matching to construct as close to an apples-to-apples comparison of private and public insurance as possible, we cannot rule out unmeasured confounding. One important policy difference that could lead to confounding is that Medicaid beneficiaries can enroll at the time of an acute episode, whereas Marketplace enrollees cannot. However, the pattern of our findings was similar even when excluding those whose first recorded claim was an ED visit or hospital stay. Moreover, we view our analysis as a major improvement in rigor over the existing literature. Although previous studies have explored differences between public and private insurance using self-reported survey data^10,11,32,33,35^ or administrative records lacking individual-level measures of socioeconomic status,^8,9^ our study uses detailed income information, rich claims data, and a design that minimized confounding by clinical differences and socioeconomic status.

## Conclusions

 This cross-sectional propensity score–matched study found important differences between Medicaid and Marketplace insurance. Medicaid coverage was associated with more ED visits and fewer office visits and prescriptions than Marketplace coverage, which may reflect greater difficulty accessing outpatient care and lower cost-sharing barriers to ED care in Medicaid. Medicaid coverage was substantially less costly to beneficiaries and society, whereas findings on quality of care were mixed. These results have important implications for policy makers considering alternative public and private approaches to coverage expansion.

## Notes

Supplement. **eMethods.** Sample Construction, Propensity Score Matching, Main Regression Models, Medicaid Price-Normalized Cost, Clinical Quality Measures, and Modified NYU Emergency Department Visit Algorithm **eTable 1.** Differences in Coverage, Utilization, Costs, and Quality Between Those Eligible for Medicaid vs Marketplace Insurance, by Year **eTable 2.** Differences in Coverage, Utilization, Costs, and Quality Between Those Eligible for Medicaid vs Marketplace Insurance, Excluding Claims From the First Month of Coverage **eTable 3.** Differences in Coverage, Utilization, Costs, and Quality Between Those Eligible for Medicaid vs Marketplace Insurance, Excluding Individuals Whose First Claim Was in the Emergency Department or Hospital **eTable 4.** Differences in Type of Emergency Department Visits Between Those Eligible for Medicaid vs Marketplace Insurance **eTable 5.** Descriptive Statistics for the Narrow Income vs Broader Income Propensity Score-Matched Samples **eTable 6.** Differences in Coverage, Utilization, Costs, and Quality Between Those Eligible for Medicaid vs Marketplace Insurance, for Broader Income Sample **eTable 7.** Differences in Secondary Measures of Health Care Quality Between Those Eligible for Medicaid vs Marketplace Insurance **eTable 8.** Comparison Between Colorado and Other States on Economic and Policy Features
eReferences
