# Supplementary material for: Comparison of Utilization, Costs, and Quality of Medicaid vs Subsidized Private Health Insurance for Low-Income Adults
Source: JAMA Netw Open. 2021 Jan 5;4(1):e2032669. doi: 10.1001/jamanetworkopen.2020.32669 (PMC9377505; doi:10.1001/jamanetworkopen.2020.32669)
Supplement: Supplement. — eMethods. Sample Construction, Propensity Score Matching, Main Regression Models, Medicaid Price-Normalized Cost, Clinical Quality Measures, and Modified NYU Emergency Department Visit Algorithm eTable 1. Differences in Coverage, Utilization, Costs, and Quality Between Those Eligible for Medicaid vs Marketplace Insurance, by Year eTable 2. Differences in Coverage, Utilization, Costs, and Quality Between Those Eligible for Medicaid vs Marketplace Insurance, Excluding Claims From the First Month of Coverage eTable 3. Differences in Coverage, Utilization, Costs, and Quality Between Those Eligible for Medicaid vs Marketplace Insurance, Excluding Individuals Whose First Claim Was in the Emergency Department or Hospital eTable 4. Differences in Type of Emergency Department Visits Between Those Eligible for Medicaid vs Marketplace Insurance eTable 5. Descriptive Statistics for the Narrow Income vs Broader Income Propensity Score-Matched Samples eTable 6. Differences in Coverage, Utilization, Costs, and Quality Between Those Eligible for Medicaid vs Marketplace Insurance, for Broader Income Sample eTable 7. Differences in Secondary Measures of Health Care Quality Between Those Eligible for Medicaid vs Marketplace Insurance eTable 8. Comparison Between Colorado and Other States on Economic and Policy Features eReferences [file jamanetwopen-e2032669-s001.pdf]

Supplementary Online Content

Allen H, Gordon SH, Lee D, Bhanja A, Sommers BD. Comparison of utilization, costs, and quality of Medicaid vs subsidized private health insurance for low-income adults. *JAMA Netw Open*. 2021;4(1):e2032669. doi:10.1001/jamanetworkopen.2020.32669

eMethods. Sample Construction, Propensity Score Matching, Main Regression Models, Medicaid Price-Normalized Cost, Clinical Quality Measures, and Modified NYU Emergency Department Visit Algorithm

eTable 1. Differences in Coverage, Utilization, Costs, and Quality Between Those Eligible for Medicaid vs Marketplace Insurance, by Year

eTable 2. Differences in Coverage, Utilization, Costs, and Quality Between Those Eligible for Medicaid vs Marketplace Insurance, Excluding Claims From the First Month of Coverage

eTable 3. Differences in Coverage, Utilization, Costs, and Quality Between Those Eligible for Medicaid vs Marketplace Insurance, Excluding Individuals Whose First Claim Was in the Emergency Department or Hospital

eTable 4. Differences in Type of Emergency Department Visits Between Those Eligible for Medicaid vs Marketplace Insurance

eTable 5. Descriptive Statistics for the Narrow Income vs Broader Income Propensity-Score Matched Samples

eTable 6. Differences in Coverage, Utilization, Costs, and Quality Between Those Eligible for Medicaid vs Marketplace Insurance, for Broader Income Sample

eTable 7. Differences in Secondary Measures of Health Care Quality Between Those Eligible for Medicaid vs Marketplace Insurance

eTable 8. Comparison Between Colorado and Other States on Economic and Policy Features

eReferences

This supplementary material has been provided by the authors to give readers additional information about their work.

© 2021 Allen H et al. *JAMA Network Open.*

eMethods. Sample Construction, Propensity Score Matching, Main Regression Models, Medicaid Price-Normalized Cost, Clinical Quality Measures, and Modified NYU Emergency Department Visit Algorithm

We obtained a dataset merging comprehensive claims data with income eligibility data for Medicaid and Marketplace enrollees in 2014-2015, through a data-use agreement with the Colorado Department of Health Care Policy and Financing (HCPF, which oversees the state's Medicaid program); the Connect for Health Colorado (C4HCO, which is the state's health insurance marketplace); and the Center for Improving Value in Health Care (CIVHC, which manages Colorado's All Payer Claims Database, or CO APCD).

Over the study period, 11 health insurance plans operated in the Colorado Marketplace, excluding dental insurance plans. The CO APCD includes six of these, while two plans Colorado HealthOP and BEST Life and Health Insurance Company, left the Marketplace in 2014 and ceased submitting claims. We assessed the share of Marketplace enrollees captured by our dataset, by comparing the sample size in our data files to the publicly-reported totals of individuals in the Colorado Marketplace. Our 2014 sample contained 64,707 Marketplace individuals with income data and FPL < 400%, compared to approximately 74,900 individuals who enrolled in C4CHO and received subsidies in 2014 (59% of the 127,000 reported by C4HCO's official statistics (https://connectforhealthco.com/wp-content/uploads/2014/05/FINAL-data-open-enrollment-report-4-14-141.pdf). For 2015, the comparable numbers were 67,431 Marketplace individuals with income data and FPL < 400% and 76,500 who enrolled in C4CHO and received subsidies in 2015 (http://connectforhealthco.wpengine.netdna-cdn.com/wp-content/uploads/2015/03/2014-OE2-

© 2021 Allen H et al. *JAMA Network Open.*

Report.pdf). This indicates our sample contains data for 86.3% and 88.1% of the state's subsidized Marketplace population for 2014 and 2015, respectively.

The unit of observation was the person-year. Individuals were included in the dataset for either year (2014 or 2015) in which they had at least one month of Medicaid or Marketplace coverage. For example, if an individual first enrolled in Medicaid in July of 2015, they would be included in the dataset for 2015, but not in 2014. Likewise, if an individual enrolled in Marketplace as of January of 2014, but dis-enrolled in July 2014 and didn't return, they would be included for 2014 but not 2015.

The CO APCD includes comprehensive data on enrollment; utilization and payments for outpatient, inpatient, and prescription drug claims; and some information on beneficiary demographics. We also obtained time-stamped family income data, as a percentage of FPL. The Medicaid sample was limited to childless adults and parents ages 19-64 who qualified for Medicaid under the ACA expansion. Our sample excluded women whose diagnosis codes indicates a pregnancy during the enrollment year. We also omitted from our sample those with incomes less than 75% of FPL, since the state's disability-related pathway to Medicaid extends through 74% of FPL. Above that income threshold, adults in the state (unless they are pregnant) are generally only eligible for subsidized coverage via the ACA's provisions – i.e. Medicaid expansion or Marketplace coverage. Medicaid data were directly matched to the CO APCD using a common identification number, whereas Marketplace data required a probabilistic match. We tested two approaches, either limiting the sample only to exact Marketplace matches by name and birthdate, or using a fuzzy match algorithm developed by the state Exchange in which we kept all observations with a cosine similarity of 0.6 or greater, which roughly corresponds to exact birthdate match and similar names, or matching names with a birthdate off by a single

© 2021 Allen H et al. *JAMA Network Open.*

digit. Our primary model included these inexact but probabilistic matches, but results were quite similar when we limited the sample to exact matches.

Income was based on the first reported value within each calendar year. While many individuals only reported income at the time of their initial enrollment for coverage, some individuals in Medicaid reported monthly changes in income during the course of the year. For Marketplace enrollees, there is only a single FPL measure per year; we attributed this measure to their first month of Marketplace enrollment, consistent with an intent-to-treat analysis of eligibility. We added 5 percentage-points of FPL to income for all Medicaid enrollees in our sample, given the ACA's statutory income disregard equal to this amount. This placed the key eligibility transition point at 138% of FPL.

Propensity Score Matching

Our propensity score model was based on a logistic regression of the following form, estimated separately for the 2014 and 2015 samples, (i.e. 2014 Medicaid observations were matched to 2014 Marketplace observations) for those with incomes between 134% and 143% of the FPL.

$$Logit(Marketplace\text{-}Eligible_{it}) = \beta_0 + \beta_1 X_{it} + \beta_2 Elixhauser_{it} + \beta_3 ChronicConditions_{it} + \beta_4 Rural_i + \varepsilon_i \qquad \text{Equation (1)}$$

The predicted value for this model was then used as the propensity score for having an income between 139-143% of FPL, and we implemented a 1:1 nearest neighbor match with the 134-138% FPL sample. We used a caliper of 0.2 of the standard deviation, and all of propensity score matches satisfied this criterion. Applying a narrower caliper of 0.02 of the standard deviation resulted in dropping 9 of the propensity score matches (18 individual observations)

© 2021 Allen H et al. *JAMA Network Open.*

from the sample, and produced very similar overall results. Descriptive statistics and standardized mean differences were then calculated for each covariate, with a threshold ≤ 0.1 used to indicate adequate balance.

Main Regression Models

Our regression analysis used Generalized Linear Models, using the distributions and link functions described in the main methods of the paper. The equation for coverage outcomes was as follows:

$$MonthsCoverage_{it} = \beta_0 + \beta_1\, Marketplace\text{-}Eligible_{it} + \beta_2\, X_{it} + \beta_3\, Elixhauser_{it} + \beta_4\, ChronicConditions_{it} + \mu\, Year_t + \Omega\, 3\text{-}Digit\_Zip_i + \varepsilon_i \qquad \text{Equation (2)}$$

where $MonthsCoverage_{it}$ is the months of coverage in the prior year with either Medicaid or Marketplace insurance, i indexes the individual and t the year. X_{it} is a vector of demographics (sex and age group), *Elixhauser* is the Elixhauser comorbidity score, and *ChronicConditions* is a vector of indicator variables for the five most chronic conditions in our sample. μ is a vector of year fixed effects (for 2014 vs. 2015), and Ω is a vector of area fixed effects at the level of the 3-digit zip code. We created a residual category for the 2.84% of our sample that did not have a three-digit zip code or resided in a zip-code with fewer than 1,000 people.

The coefficient of interest is β_1, which measures the outcome difference associated with having an initial income above 138% of FPL, making that person eligible for Marketplace coverage, rather than Medicaid.

The regression used Huber-White robust standard errors clustered at the level of the individual, to account for repeated measures for those appearing separately in both years of the dataset.

The equation for utilization, cost, and quality outcomes was as follows:

© 2021 Allen H et al. *JAMA Network Open.*

$$Y_{it} = \beta_0 + \beta_1 \, Marketplace\text{-}Eligible_{it} + \beta_2 \, X_{it} + \beta_3 \, Elixhauser_{it} + \beta_4 \, ChronicConditions_{it} + \beta_5 \, MonthsCoverage_{it} + \mu \, Year_t + \Omega \, 3\text{-}Digit\_Zip_i + \varepsilon_i$$

Equation (3)

The only differences between Equations 2 and 3 were the outcome variable, and the addition of Months of Coverage as an independent variable.

For secondary quality outcomes, several of which had much smaller condition-specific samples, we used a more parsimonious version of Equation 3 to reduce the risk of overfitting and/or dropping observations due to perfectly predicting outcomes. This equation replaced the three digit zip code fixed effects with an indicator for urban vs. rural residence, and only used the overall Elixhauser score but not specific condition indicators:

$$Y_{it} = \beta_0 + \beta_1 \, Marketplace\text{-}Eligible_{it} + \beta_2 \, X_{it} + \beta_3 \, Elixhauser_{it} + \beta_4 \, Rural_i + \beta_5 \, MonthsCoverage_{it} + \mu \, Year_t + \varepsilon_i$$

Equation (4)

All coefficients were then converted into adjusted outcome estimates using the “margins” command in Stata for *Marketplace-Eligible*, which provides separate sample-wide marginal outcomes for the *Marketplace-Eligible* population and the *Medicaid-Eligible* population, using each observations’ actual covariates and the coefficients from the relevant regression model described above, except for the coverage measures, which used the margins “at means” option (using the means of the covariates) – see Table 2 footnotes for details.

Medicaid Price-Normalized Cost

We analyzed Medicaid price-normalized costs to facilitate comparisons between the overall health care utilization across coverage types, after removing the impact of differential prices for health care services in the difference insurance plans. To create this outcome, we calculated the mean costs of CPT procedure codes found in claims in the Medicaid database, and

© 2021 Allen H et al. *JAMA Network Open.*

then applied the mean Medicaid cost per procedure to all claims (whether they were Medicaid or Marketplace) to derive the price-normalized cost for each individual in our sample.

The initial average costs of CPT procedure codes were calculated from claims with only a single unique CPT code, which accounted for 8,157 CPT codes. For the remaining codes found only in multiple-CPT claims, we iteratively calculated the average costs of the unknown CPT codes by subtracting total claim costs by the costs calculated from known codes. For the claims with “j” total CPT codes with “j-1” CPT codes with a calculated cost, where “j” is an integer, the remainder provides an estimate of the unknown CPT code’s cost. These averages were then stored and used in the next iteration to calculate remaining prices. This process had reached completion with a total of six iterations. This process allowed us to calculate prices for 10,051 CPT codes, leaving 922 unmatched codes, which represented only 0.07% of claims, for which we set the effective price to $0.

These mean Medicaid prices were then assigned to all Medicaid ad Marketplace claims via CPT codes in order to calculate the price-normalized costs per enrollee. Negative yearly enrollee costs (which resulted from the above iteration for multiple CPT codes) affected 0.05% of the sample and were truncated to zero in our final analysis.

Clinical Quality Measures

We created binary indicators for the following measures of high-value care:

- ***Mammography*** – The denominator included all women ages 50-64, based on current HEDIS guidelines,[1] and the numerator included anyone with a claim for a mammogram during the calendar year.

© 2021 Allen H et al. *JAMA Network Open.*

- ***Influenza Vaccination*** **–** The denominator included all adults aged 19-64, based on current National Quality Forum guidelines,[2] and the numerator included anyone with a claim for influenza immunization during the calendar year.
- ***Chlamydia Screening*** **–** The denominator included all non-pregnant women aged 19-24, based on current HEDIS guidelines (note that the claims data do not allow us to distinguish between women who are and are not sexually active),[3] and the numerator included anyone with a claim for a chlamydia test during the calendar year.
- ***Beta Blockers in Patients with Coronary Artery Disease*** **–** The denominator included all adults with coronary artery disease, and the numerator included anyone with a prescription claim for beta blockers during the calendar year.[4] While the benefit of indefinite therapy with beta blockers in uncomplicated CAD remains unclear and an area of clinical investigation, this remains a commonly-used quality measure that can be evaluated with claims data.
- ***Statin Use in Diabetics Over Age 40 and Patients with Atherosclerotic Disease*** **–** The denominator included all adults aged over 40 with atherosclerotic disease, and the numerator included anyone with a prescription claim for statin during the calendar year.[5]
- ***Hemoglobin A1c Testing in Patients with Diabetes*** **–** The denominator included all adults with diabetes, and the numerator included anyone with a claim for hemoglobin A1c testing during the measurement year.[1]
- ***Urine Microalbumin Testing in Patients with Diabetes*** **–** The denominator included all adults with diabetes, and the numerator included anyone with a claim for urine microalbumin during the measurement year.[6]

© 2021 Allen H et al. *JAMA Network Open.*

- ***Annual Eye Exam in Patients with Diabetes* –** The denominator included all adults with diabetes, and the numerator included anyone with a claim for eye exam during the measurement year.[7]

We also created binary indicators for the following measures of low-value care, identified in prior research (Barnett, et al. 2017)[8]:

- ***Advanced Imaging for Uncomplicated Back Pain < 6 Weeks Duration* –** The denominator included all individuals with a claim including a diagnosis code for back pain, excluding those with fever, weight loss, neurologic symptoms, cancer, fracture, myelopathy, prior back surgery, or a prior visit at least two months earlier for back pain.[8] The numerator included a claim for CT or MRI of the back within the month or month following the initial visit.
- ***Advanced Imaging for Uncomplicated Headache* –** The denominator included all individuals with a claim including a diagnosis code for headache, excluding those with HIV, pregnancy, neurologic symptoms, cancer, fracture, or epilepsy.[8] The numerator included a claim for CT or MRI of the head within the month or month following the initial visit.
- ***Narcotic Prescription for Headache* –** The denominator included all individuals with a claim including a diagnosis code for headache, excluding those with HIV, pregnancy, neurologic symptoms, cancer, fracture, or epilepsy.[8] The numerator included a prescription claim for any narcotics for the same month of the initial visit.
- ***Antibiotic Prescription for Upper Respiratory Infection* –** The denominator included all individuals with a claim for an acute upper respiratory infection, excluding chronic

© 2021 Allen H et al. *JAMA Network Open.*

respiratory illness, cancer, HIV, or sexually transmitted infections.[8] The numerator included a prescription claim for antibiotics for the same month of the intial visit.

Modified NYU Emergency Department Visit Algorithm

We have classified emergency department visits according to the updated Emergency Department (ED) algorithm from Johnston et al. (2017), based on the original New York University ED visit algorithm. This approach assigns probabilistic weights to four classifications, which we summed across all ED visits to generate individual-level yearly total counts of emergency department visits by severity category. The categories and corresponding definitions were:

- *Non-emergent* – "Immediate medical care was not required within 12 hours."
- *Emergent but primary care treatable* – "Treatment was required within 12 hours, but care could have been provided effectively and safely in a primary care setting. The complaint did not require continuous observation, and no procedures were performed or resources used that are not available in a primary care setting."
- *Emergent but preventable* – "Emergency department care was required based on the complaint or procedures performed/resources used, but the emergent nature of the condition was potentially preventable/avoidable if timely and effective ambulatory care had been received during the episode of illness."
- *Emergent and not preventable* – "Emergency department care was required and ambulatory care treatment could not have prevented the condition."

eTable 1. Differences in Coverage, Utilization, Costs, and Quality Between Those Eligible for Medicaid vs Marketplace Insurance, by Year

OUTCOME	2014					2015				
	Medicaid-Eligible (Income 134% to <=138%)		Marketplace-Eligible (Income >138% to <=143%)		Public vs. Private	Medicaid-Eligible, (Income 134% to <=138%)		Marketplace-Eligible (Income >138% to <=143%)		Public vs. Private
	N = 2178		N = 2178			N = 1913		N = 1913		
	Adjusted Mean	95% CI	Adjusted Mean	95% CI	P value	Adjusted Mean	95% CI	Adjusted Mean	95% CI	P value
**COVERAGE**										
Months of Medicaid or Marketplace Coverage	8.68	8.52, 8.83	8.52	8.38, 8.65	0.13	9.24	9.08, 9.40	8.51	8.32, 8.70	<0.001
Months of Medicaid Coverage	8.52	8.31, 8.72	1.68	1.54, 1.81	<0.001	9.28	9.07, 9.49	3.21	3.01, 3.42	<0.001
Months of Marketplace Coverage	0.52	0.43, 0.61	6.80	6.58, 7.02	<0.001	0.23	0.17, 0.29	5.53	5.24, 5.81	<0.001
**UTILIZATION (per year)**										
Outpatient visits	1.85	1.73, 1.97	2.29	2.14, 2.43	<0.001	1.58	1.47, 1.69	2.14	1.99, 2.28	<0.001
Emergency Department visits	0.66	0.55, 0.78	0.38	0.31, 0.45	<0.001	0.45	0.40, 0.51	0.34	0.29, 0.39	0.002
Prescription drug fills	8.93	8.02, 9.84	8.35	7.47, 9.22	0.26	5.86	5.20, 6.51	8.26	7.37, 9.14	<0.001
Hospitalizations	0.040	0.023, 0.057	0.029	0.017, 0.041	0.15	0.020	0.012, 0.028	0.024	0.016, 0.033	0.45
**COST**										
Total health care costs ($)	2,841	1,588, 4,095	5,053	3,050, 7,055	<0.001	2,289	1,611, 2,966	4,115	2,932, 5,299	<0.001
Out-of-pocket costs ($)§	52	27, 77	600	316, 884	<0.001	39	11, 68	706	182, 1,230	<0.001
Normalized spending, using mean Medicaid prices ($)†	1,689	1,211, 2,166	1,959	1,439, 2,479	0.09	1,333	1,104, 1,562	1,393	1,177, 1,609	0.65
**QUALITY**										
Ambulatory Care Sensitive Hospitalizations	0.011	-0.009, 0.030	0.006	-0.006, 0.018	0.28	0.005	0.002, 0.008	0.002	0.000, 0.004	0.14

© 2021 Allen H et al. *JAMA Network Open.*

Notes:
Data are from the Colorado All Payer Claims Database, linked to income data from Medicaid and Marketplace eligibility files. Sample contains propensity-score matched adults ages 19-64, with incomes between 134% and 143% of FPL (N=8,182). Models adjusted for age, sex, Elixhauser comorbidity index (overall score and top five conditions), year, and three-digit zip code; utilization, cost, and quality outcomes also adjusted for total months of Medicaid or Marketplace coverage. Coverage, utilization, and quality outcomes were analyzed using a generalized linear model (GLM) with a negative binomial distribution. Costs outcomes were analyzed using a GLM with a gamma distribution and log link, with outcomes in 2015-inflation adjusted terms. All regression results were converted to adjusted means based on the observed distribution of covariates using the *margins* command in Stata, other than for coverage outcomes. Coverage outcomes were assessed using *margins* at covariate means, due to totaling errors with the *margins* command at the observed distribution (i.e. total months of coverage < months Medicaid).
95% CI = "95% Confidence Interval"
§ Out-of-pocket costs are the charged amount; the dataset does not indicate whether patients paid the required amount.
† This outcome was calculated using mean Medicaid price per service provided, to provide an aggregate measure of health care resources consumed but using the same price regardless of the person's type of health insurance. See Appendix methods for further details.

© 2021 Allen H et al. *JAMA Network Open.*

eTable 2.

Differences in Utilization, Costs, and Quality Between Those Eligible for Medicaid vs Marketplace Insurance, Excluding Claims From the First Month of Coverage

OUTCOME	Medicaid-Eligible (Income 134% to <=138%)		Marketplace-Eligible (Income >138% to <=143%)		Public vs. Private Coverage
	N = 4091		N = 4091		
	Adjusted Mean	95% CI	Adjusted Mean	95% CI	P value
**UTILIZATION (per year)**					
Outpatient visits	1.56	(1.48, 1.64)	1.99	(1.89,2.09)	<0.001
Emergency Department visits	0.45	(0.40, 0.50)	0.32	(0.28, 0.35)	<0.001
Prescription drug fills	6.77	(6.22, 7.31)	7.54	(6.93, 8.15)	0.03
Hospitalizations	0.026	(0.017, 0.034)	0.025	(0.017, 0.033)	0.95
**COST**					
Total health care costs ($)	2,317	(1,527, 3,108)	4,456	(3,113, 5,798)	<0.001
Out-of-pocket costs ($)§	50	(25, 74)	660	(336, 984)	<0.001
Normalized spending, using mean Medicaid prices ($)†	1,283	(1,038, 1,528)	1,557	(1,303, 1,811)	0.008
**QUALITY**					
Ambulatory Care Sensitive Hospitalizations	0.005	(0.000, 0.010)	0.003	(0.000, 0.005)	0.14

Notes:
Sample excludes all utilization from an individual's first month of coverage in the calendar year.
Data are from the Colorado All Payer Claims Database, linked to income data from Medicaid and Marketplace eligibility files. Sample contains propensity-score matched adults ages 19-64, with incomes between 134% and 143% of FPL (N=8,182). Models adjusted for age, sex, Elixhauser comorbidity index (overall score and top five conditions), year, three-digit zip code, and total months of Medicaid or Marketplace coverage. Utilization and quality outcomes were analyzed using a generalized linear model (GLM) with a negative binomial distribution. Costs outcomes were analyzed using a GLM with a gamma distribution and log link, with outcomes in 2015-inflation adjusted terms. All regression results were converted to adjusted means based on the observed distribution of covariates using the *margins* command in Stata.
95% CI = "95% Confidence Interval"
§ Out-of-pocket costs are the charged amount; the dataset does not indicate whether patients paid the required amount.
† This outcome was calculated using mean Medicaid price per service provided, to provide an aggregate measure of health care resources consumed but using the same price regardless of the person's type of health insurance. See Appendix methods for further details.

© 2021 Allen H et al. *JAMA Network Open.*

eTable 3. Differences in Coverage, Utilization, Costs, and Quality Between Those Eligible for Medicaid vs Marketplace Insurance, Excluding Individuals Whose First Claim Was in the Emergency Department or Hospital

**OUTCOME**	**Model Coefficient**	**Medicaid-Eligible (Income 134% to <=138%)**		**Marketplace-Eligible (Income >138% to <=143%)**		**Public vs. Private Coverage**
		**n = 4000**		**n = 4000**		
		**Adjusted Mean**	**95% CI**	**Adjusted Mean**	**95% CI**	**P value**
**UTILIZATION (per year)**						
Outpatient visits	0.27	1.65	(1.57, 1.73)	2.16	(2.05, 2.26)	<0.001
Emergency Department visits	-0.34	0.39	(0.35, 0.43)	0.28	(0.25, 0.31)	<0.001
Prescription drug fills	0.14	6.85	(6.33, 7.37)	7.90	(7.29, 8.52)	0.004
Hospitalizations	-0.05	0.024	(0.015, 0.032)	0.023	(0.014, 0.031)	0.85
**COST**						
Total health care costs ($)	0.70	2,003	(1,413, 2,593)	4,030	(3,029, 5,031)	<0.001
Out-of-pocket costs ($)§	2.66	42	(24, 60)	594	(355, 833)	<0.001
Normalized spending, using mean Medicaid prices ($)†	0.18	1,279	(1,030, 1,527)	1,527	(1,268, 1,786)	0.015
**QUALITY**						
Ambulatory Care Sensitive Hospitalizations	-0.47	0.003	(0.001, 0.006)	0.002	(0.001, 0.003)	0.36

Notes:
Sample contains propensity-score matched adults ages 19-64, with incomes between 134% and 143% of FPL, and excludes all individuals whose first recorded claim was an ED or hospital inpatient claim during their first month of coverage in the calendar year (N=8,000).
Data are from the Colorado All Payer Claims Database, linked to income data from Medicaid and Marketplace eligibility files. Models adjusted for age, sex, Elixhauser comorbidity index (overall score and top five conditions), year, three-digit zip code, and total months of Medicaid or Marketplace coverage. Utilization and quality outcomes were analyzed using a generalized linear model (GLM) with a negative binomial distribution. Costs outcomes were analyzed using a GLM with a gamma distribution and log link, with outcomes in 2015-inflation adjusted terms. All regression results were converted to adjusted means based on the observed distribution of covariates using the *margins* command in Stata.
95% CI = "95% Confidence Interval"
§ Out-of-pocket costs are the charged amount; the dataset does not indicate whether patients paid the required amount.
† This outcome was calculated using mean Medicaid price per service provided, to provide an aggregate measure of health care resources consumed but using the same price regardless of the person's type of health insurance. See Appendix methods for further details.

© 2021 Allen H et al. *JAMA Network Open.*

eTable 4. Differences in Type of Emergency Department Visits Between Those Eligible for Medicaid vs Marketplace Insurance

OUTCOME	Model Coefficient (Incident Rate Ratios)	Medicaid-Eligible (Income 134% to <=138%)		Marketplace-Eligible (Income >138% to <=143%)		Public vs. Private Coverage
		N = 4091		N = 4091		
		Adjusted Mean	95% CI	Adjusted Mean	95% CI	P value
**Emergency Department Visits (per year)**						
Overall	0.62	0.56	(0.50, 0.62)	0.36	(0.32, 0.40)	<0.001
Emergent, ED Care Needed, Not Preventable	0.66	0.11	(0.09, 0.13)	0.07	(0.06, 0.08)	<0.001
Emergent, ED Care Needed, Preventable	0.60	0.04	(0.03, 0.06)	0.03	(0.01, 0.04)	0.001
Emergent, Primary Care Treatable	0.60	0.13	(0.11, 0.14)	0.08	(0.07, 0.09)	<0.001
Non-Emergent	0.69	0.12	(0.10, 0.13)	0.08	(0.07, 0.09)	<0.001
Injury Related	0.72	0.11	(0.09, 0.12)	0.08	(0.06, 0.09)	0.003
Mental Health and Alcohol/Drug Related†	0.74	0.01	(0.01, 0.02)	0.01	(0.00, 0.01)	0.32
Unclassified	0.53	0.04	(0.03, 0.05)	0.02	(0.02, 0.03)	<0.001

Notes:
Data are from the Colorado All Payer Claims Database, linked to income data from Medicaid and Marketplace eligibility files. Sample contains propensity-score matched adults ages 19-64, with incomes between 134% and 143% of FPL (N=8,182). Models adjusted for age, sex, Elixhauser comorbidity index (overall score and top five conditions), year, three-digit zip code, and total months of Medicaid or Marketplace coverage. Utilization outcomes were analyzed using a generalized linear model (GLM) with a negative binomial distribution. All regression results were converted to adjusted means based on the observed distribution of covariates using the *margins* command in Stata.
† Some substance-abuse disorder claims are excluded from the APCD, based on federal regulations - see Methods for details.
95% CI = "95% Confidence Interval"

© 2021 Allen H et al. *JAMA Network Open.*

eTable 5. Descriptive Statistics for the Narrow Income vs Broader Income Propensity-Score Matched Samples

Characteristic	Propensity Score Matched Sample (Income 134%-143% of FPL)			Propensity Score Matched Sample (Income 129%-148% of FPL)		
	Medicaid Eligible (≤138% FPL)	Marketplace Eligible (>138% FPL)	SMD	Medicaid Eligible (≤138% FPL)	Marketplace Eligible (>138% FPL)	SMD
**Sample size (N)**	4,091	4,091		8,641	8,641	
Average Income (% FPL)	136%	141%		134%	144%	
MATCHING VARIABLES						
Age (years)	42.8 (13.6)	42.7 (13.9)	0.004	42.3 (13.5)	42.3 (13.8)	0.003
–19-25 (%), (n)	563 (13.8)	569 (13.9)	0.004	1,148 (13.3)	1,155 (13.4)	0.002
–26-34	842 (20.6)	844 (20.6)	0.001	1,936 (22.4)	1,931 (22.4)	0.001
–35-44	691 (16.9)	686 (16.8)	0.003	1,526 (17.7)	1,512 (17.5)	0.004
–45-54	866 (21.2)	857 (21.0)	0.005	1,768 (20.5)	1,775 (20.6)	0.002
–55-64	1,129 (27.6)	1,135 (27.7)	0.003	2,263 (26.2)	2,268 (26.3)	0.001
Sex						
–Male	1,859 (45.4)	1,861 (45.5)	0.003	3,861 (44.7)	3,879 (44.9)	0.005
–Female	2,230 (54.5)	2,229 (54.5)	0.002	4,778 (55.3)	4757 (55.1)	0.005
Rural Area of Residence	458 (11.2)	458 (11.2)	0.000	927 (10.7)	933 (10.8)	0.002
Elixhauser Comorbidity Index	0.22	0.25	0.013	0.20	0.22	0.014
Most Common Chronic Conditions						
–Hypertension	178 (4.4)	179 (4.4)	0.001	344 (4.0)	361 (3.2)	0.01
–Depression	170 (4.2)	181 (4.4)	0.016	374 (4.3)	365 (4.2)	0.006
–Chronic Obstructive Pulmonary Disease (COPD)	127 (3.1)	129 (3.2)	0.004	252 (2.9)	260 (3.0)	0.005
–Hypothyroidism	114 (2.8)	113 (2.8)	0.004	247 (2.9)	244 (2.8)	0.003
–Diabetes	165 (4.0)	162 (4.0)	0.004	327 (3.8)	335 (3.9)	0.004

Notes: Sample from Colorado All Payer Claims Database, linked to income data from Medicaid and Marketplace eligibility files.
FPL = "Federal Poverty Level."
SMD = Standardized Mean Difference (absolute value of difference in means / standard deviation)

© 2021 Allen H et al. *JAMA Network Open.*

eTable 6.

Differences in Coverage, Utilization, Costs, and Quality Between Those Eligible for Medicaid vs Marketplace Insurance, for Broader Income Sample

OUTCOME	Medicaid-Eligible (Income ≤ 138% FPL)		Marketplace-Eligible (Income > 138% FPL)		Public vs. Private Coverage
	N = 8641		N = 8641		
	Adjusted Mean	95% CI	Adjusted Mean	95% CI	P value
**COVERAGE**					
Months of Medicaid or Marketplace Coverage	8.99	(8.92, 9.07)	8.43	(8.34, 8.51)	<0.001
Months of Medicaid Coverage	8.75	(8.65, 8.84)	2.29	(2.21, 2.38)	<0.001
Months of Marketplace Coverage	0.35	(0.31, 0.39)	5.57	(5.44, 5.69)	<0.001
**UTILIZATION (per year)**					
Outpatient visits	1.74	(1.68, 1.80)	2.15	(2.08, 2.22)	<0.001
Emergency Department visits	0.53	(0.49, 0.57)	0.35	(0.32, 0.37)	<0.001
Prescription drug fills	7.41	(7.04, 7.79)	7.85	(7.44, 8.25)	0.09
Hospitalizations	0.026	(0.020, 0.032)	0.028	(0.022, 0.034)	0.58
**COST**					
Total health care costs ($)	2,047	(1,809, 2,285)	3,861	(3,405, 4,318)	<0.001
Out-of-pocket costs ($)§	38	(31, 45)	347	(288, 407)	<0.001
Normalized spending, using mean Medicaid prices ($)†	1,328	(1,198, 1,458)	1,482	(1,340, 1,624)	0.08
**QUALITY**					
Ambulatory Care Sensitive Hospitalizations	0.007	(0.004, 0.010)	0.005	(0.003, 0.007)	0.21

Notes:
Data are from the Colorado All Payer Claims Database, linked to income data from Medicaid and Marketplace eligibility files. Sample contains propensity-score matched adults ages 19-64, with incomes between 129% and 148% of FPL (N=17,282). Models adjusted for age, sex, Elixhauser comorbidity index (overall score and top five conditions), year, and three-digit zip code; utilization, cost, and quality outcomes also adjusted for total months of Medicaid or Marketplace coverage. Coverage, utilization, and quality outcomes were analyzed using a generalized linear model (GLM) with a negative binomial distribution. Costs outcomes were analyzed using a GLM with a gamma distribution and log link, with outcomes in 2015-inflation adjusted terms. All regression results were converted to adjusted means based on the observed distribution of covariates using the *margins* command in Stata, other than for coverage outcomes. Coverage outcomes were assessed using *margins* at covariate means, due to totaling errors with the *margins* command at the observed distribution (i.e. total months of coverage < months Medicaid).
95% CI = "95% Confidence Interval"

© 2021 Allen H et al. *JAMA Network Open.*

§ Out-of-pocket costs are the charged amount; the dataset does not indicate whether patients paid the required amount.
† This outcome was calculated using mean Medicaid price per service provided, to provide an aggregate measure of health care resources consumed but using the same price regardless of the person's type of health insurance. See Appendix methods for further details.

© 2021 Allen H et al. *JAMA Network Open.*

eTable 7.
Differences in Secondary Measures of Health Care Quality
Between Those Eligible for Medicaid v. Marketplace Insurance

OUTCOME	Sample Size	Medicaid-Eligible (Income ≤ 138% FPL)		Marketplace-Eligible (Income > 138% FPL)		Public vs. Private Coverage	
		Adjusted Mean	95% CI	Adjusted Mean	95% CI	P value	Adjusted P value*
**HIGH VALUE CARE**							
Mammogram, Women 50-64	3,663	0.18	(0.16, 0.20)	0.22	(0.20, 0.24)	0.007	0.03
Chlamydia Testing, Women 19-24	999	0.14	(0.11, 0.17)	0.22	(0.19, 0.26)	0.002	0.008
Hemoglobin A1c Test, Patients with Diabetes	662	0.72	(0.67, 0.77)	0.86	(0.82, 0.90)	<0.001	<0.001
Urine Microalbumin Test, Patients with Diabetes	662	0.38	(0.33, 0.43)	0.50	(0.45, 0.55)	0.002	0.009
Eye Exam, Patients with Diabetes	662	0.20	(0.16, 0.25)	0.24	(0.19, 0.29)	0.27	0.48
Beta-Blocker Use, Patients with Coronary Artery Disease	131	0.68	(0.58, 0.79)	0.47	(0.33, 0.61)	0.03	0.09
Statin Use, Diabetics Over age 40 and Patients with Atherosclerotic Disease	822	0.52	(0.47, 0.57)	0.51	(0.47, 0.56)	0.90	0.91
Influenza Vaccine	17,282	0.04	(0.04, 0.04)	0.05	(0.05, 0.06)	<0.001	0.002
**LOW VALUE CARE**							
Antibiotics for Upper Respiratory Infection	853	0.33	(0.29, 0.38)	0.34	(0.30, 0.39)	0.75	0.99
CT/MRI for Back Pain < 6 Weeks Duration	1,086	0.34	(0.31, 0.38)	0.36	(0.32, 0.40)	0.61	0.98
CT/MRI for Headache	407	0.32	(0.26, 0.39)	0.31	(0.25, 0.37)	0.81	0.96
Narcotic Prescription for Headache	407	0.19	(0.13, 0.24)	0.18	(0.13, 0.23)	0.81	0.81

Notes:
Data are from the Colorado All Payer Claims Database, linked to income data from Medicaid and Marketplace eligibility files. Sample contains propensity-score matched adults ages 19-64, with incomes between 129% and 148% of FPL (N=17,282). Models adjusted for age, sex, Elixhauser comorbidity index, urban vs. rural residence, and total months of Medicaid or Marketplace coverage. All outcomes were analyzed using a generalized linear model (GLM) with a Binomial distribution and logistic link. All regression results were converted to adjusted means based on the observed distribution of covariates using the *margins* command in Stata.
"95% CI = "95% Confidence Interval
* These p-values were adjusted according to the family-wise error rate, using the Westfall and Young (1993) free step-down resampling approach, to account for multiple outcomes within each category.

© 2021 Allen H et al. *JAMA Network Open.*

eTable 8. Comparison Between Colorado and Other States on Economic and Policy Features

Domain	Colorado	United States	Colorado State Rank
Uninsured rate, adults 19-64 (2015)[11]	11%	13%	28/50
Median income[12]	$65,458	$57,652	12/50
Medicaid managed care penetration[13-14]	10%	69%	13/50
Medicaid-to-Medicare fee index (all services, 2016)[15-16]	0.80	0.72	29/50
Primary care Medicaid-to-Medicare fee index (2016)[15-16]	0.84	0.66	38/50
Medicaid participation rate among non-elderly adults, pre-ACA (2009)[17]	63.8%	67.4%	37/50
Number of insurers participating in Marketplace (2015)[18]	10	6	41/50
Marketplace enrollment as a share of the potential Marketplace population (2019)[19]	25%	23%	16/50

© 2021 Allen H et al. *JAMA Network Open.*

eReferences

1. “2018 Coding Reference Guide for Primary Care.” HEDIS 2018 Coding Reference Guide for Primary Care, MVP Health Care, www.ncqa.org/hedis-quality-measurement/hedis-measures/hedis-2018 .
2. “Health and Well-Being 2015-2017: Technical Report.” National Quality Forum, http://www.qualityforum.org/Projects/h/Health_and_Well_Being_2015-2017/Final_Report.aspx.
3. “HEDIS 2017 Measure: Chlamydia Screening in Women (CHL)”, Rday Children’s Health Network, https://www.rchsd.org/documents/2018/08/chl-chlamydia-screening-in-women.pdf.
4. “Cardiovascular Conditions 2016-2017: Technical Report”, National Quality Forum, http://www.qualityforum.org/Publications/2017/02/Cardiovascular_Conditions_2016-2017.aspx.
5. “2017 MIPS Measure #438: Statin Therapy for the Prevention and Treatment of Cardiovascular Disease”, Healthmonix, http://healthmonix.com/mips_quality_measure/statin-therapy-for-the-prevention-and-treatment-of-cardiovascular-disease-measure-438/.
6. “Quality ID #119 (NQF 0062): Diabetes: Medical Attention for Nephropathy – National Quality Strategy”, American Medical Association, https://www.ncdr.com/WebNCDR/docs/default-source/pinnacle-public-documents/2018_measure_119_registry.pdf?sfvrsn=2.
7. “Quality ID #117 (NQF 0055): Diabetes: Eye Exam – National Quality Strategy Domain: Effective”, American Medical Association, https://qpp.cms.gov/docs/QPP_quality_measure_specifications/Claims-Registry-Measures/2018_Measure_117_Registry.pdf.
8. Barnett ML, Linder JA, Clark CR, Sommers BD. Low-Value Medical Services in the Safety-Net Population. JAMA Intern Med. 2017;177(6):829–837. doi:10.1001/jamainternmed.2017.0401
9. Johnston KJ, Allen L, Melanson TA, Pitts SR. A "Patch" to the NYU Emergency Department Visit Algorithm. Health Serv Res. 2017;52(4):1264–1276. doi:10.1111/1475-6773.12638
10. Billings J., Parikh N., and Mijanovich T. “Emergency Department Use: The New York Story.” Issue Brief Commonwealth Fund 2000.
11. State Health Facts, 2019. Washington DC, Kaiser Family Foundation. Available at: <https://http://www.kff.org/statedata/>
12. U.S. Census Bureau. "Fact Finder: Median Household Income in 2017 Inflation-Adjusted Dollars." Washington, DC: Census Bureau. https://factfinder.census.gov/faces/tableservices/jsf/pages/productview.xhtml?src=CF
13. Kaiser Family Foundation (KFF). Medicaid Managed Care Penetration Rates by Eligibility Group. 2019. Available from: https://www.kff.org/medicaid/state-indicator/managed-care-penetration-rates-by-eligibility-group/?currentTimeframe=0&sortModel=%7B%22colId%22:%22Location%22,%22sort%22:%22asc%22%7D
14. Kaiser Family Foundation (KFF). Total Medicaid MCO Enrollment. 2017. Available from: https://www.kff.org/other/state-indicator/total-medicaid-mco-

© 2021 Allen H et al. *JAMA Network Open.*

enrollment/?currentTimeframe=0&sortModel=%7B%22colId%22:%22Location%22,%22sort%22:%22asc%22%7D
15. Zuckerman S, L Skopec, M Epstein. 2017. Medicaid physicians fees after the ACA primary care fee bump. Available from: https://www.urban.org/research/publication/medicaid-physician-fees-after-aca-primary-care-fee-bump
16. Kaiser Family Foundation (KFF). Medicaid to Medicare Fee Index. 2016. Available from: https://www.kff.org/medicaid/state-indicator/medicaid-to-medicare-fee-index/?currentTimeframe=0&sortModel=%7B%22colId%22:%22Location%22,%22sort%22:%22asc%22%7D
17. Kenney GM, Lynch V, Haley J, Huntress M. Variation in Medicaid eligibility and participation among adults: implications for the Affordable Care Act. INQUIRY: The Journal of Health Care Organization, Provision, and Financing. 2012 Aug;49(3):231-53.
18. Kaiser Family Foundation (KFF). Number of Issuers Participating in the Individual Health Insurance Marketplaces. 2015. Available from: https://www.kff.org/other/state-indicator/number-of-issuers-participating-in-the-individual-health-insurance-marketplace/?currentTimeframe=0&sortModel=%7B%22colId%22:%22Number%20of%20Issuers%20in%202015%22,%22sort%22:%22asc%22%7D
19. Kaiser Family Foundation (KFF). Marketplace Enrollment as a Share of the Potential Marketplace Population. 2019. Available from: https://www.kff.org/health-reform/state-indicator/marketplace-plan-selections-as-a-share-of-the-potential-marketplace-population/?currentTimeframe=0&sortModel=%7B%22colId%22:%22Percent%20of%20Potential%20Marketplace%20Population%20Enrolled%22,%22sort%22:%22asc%22%7D

© 2021 Allen H et al. *JAMA Network Open.*
